# Advanced ensemble modelling of flexible macromolecules using X-ray solution scattering

**DOI:** 10.1107/S205225251500202X

**Published:** 2015-02-26

**Authors:** Giancarlo Tria, Haydyn D. T. Mertens, Michael Kachala, Dmitri I. Svergun

**Affiliations:** a European Molecular Biology Laboratory, Hamburg Outstation, c/o DESY, Notkestrasse 85, Hamburg, 22603, Germany

**Keywords:** small-angle scattering, proteins, macromolecular dynamics, unstructured biology, hybrid methods, symmetric oligomers

## Abstract

Dynamic ensembles of macromolecules mediate essential processes in biology. Understanding the mechanisms driving the function and molecular interactions of ‘unstructured’ and flexible molecules requires alternative approaches to those traditionally employed in structural biology. Small-angle X-ray scattering (SAXS) is an established method for structural characterization of biological macromolecules in solution, and is directly applicable to the study of flexible systems such as intrinsically disordered proteins and multi-domain proteins with unstructured regions. The *Ensemble Optimization Method* (*EOM*) [Bernadó *et al.* (2007 ▶). *J. Am. Chem. Soc.* **129**, 5656–5664] was the first approach introducing the concept of ensemble fitting of the SAXS data from flexible systems. In this approach, a large pool of macromolecules covering the available conformational space is generated and a sub-ensemble of conformers coexisting in solution is selected guided by the fit to the experimental SAXS data. This paper presents a series of new developments and advancements to the method, including significantly enhanced functionality and also quantitative metrics for the characterization of the results. Building on the original concept of ensemble optimization, the algorithms for pool generation have been redesigned to allow for the construction of partially or completely symmetric oligomeric models, and the selection procedure was improved to refine the size of the ensemble. Quantitative measures of the flexibility of the system studied, based on the characteristic integral parameters of the selected ensemble, are introduced. These improvements are implemented in the new *EOM* version 2.0, and the capabilities as well as inherent limitations of the ensemble approach in SAXS, and of *EOM* 2.0 in particular, are discussed.

## Abstract 2

New developments in the modelling of flexible biological macromolecules from SAXS data offer extended possibilities of using high-resolution models and provide metrics for quantitative characterization of the reconstructed ensembles.

## 1. Introduction   

Small-angle X-ray scattering (SAXS) of particles in solution is a widely used technique for the structural characterization of biological macromolecules (Svergun *et al.*, 2013 ▶; Feigin & Svergun, 1987 ▶). Perhaps one of the most exciting applications of the technique is in the field of *unstructural biology* (Tompa, 2011 ▶), where SAXS is increasingly employed to extract information from extremely challenging systems including flexible multi-domain proteins with disordered inter-domain linkers and intrinsically disordered proteins (IDPs). Flexible particles are difficult objects to study and often little is known about their structural organization or lack thereof. It is increasingly recognized that structural disorder appears to be a common feature of functional macromolecules with approximately 40% of proteins in the human genome presenting at least one disordered region (≥ 30 residues) and 25% likely to be completely disordered (Chouard, 2011 ▶), obviating to some extent the traditional dogma of structural biology: *function requires structure*. An increasing number of studies demonstrate that the absence of a stable predefined shape does not prevent biomolecules such as proteins from performing important biological functions (Rubio-Cosials *et al.*, 2011 ▶; Devarakonda *et al.*, 2011 ▶; Pérard *et al.*, 2013 ▶; Uversky *et al.*, 2008 ▶). Instead, structural plasticity is often essential for the execution of specific roles, in particular for proteins that perform both multiple and single tasks. Consequently the concept of disorder has been revisited and associated with a macromolecule’s complexity; however, the standard high-resolution approaches of structural biology are limited in their ability to characterize disordered systems. Crystallization of flexible systems for macromolecular X-ray crystallography (MX) is challenging, and the inherent size limitations (∼60 kDa) of nuclear magnetic resonance (NMR) hinder applicability. Furthermore, when applied to flexible particles electron microscopy (EM) and atomic force microscopy (AFM) often yield complex and ambiguous results. Without the requirement for crystals and without effective size limitations, SAXS in near-native solutions is becoming more and more popular for the characterization of such systems.

Qualitative and quantitative scattering studies can be conducted on disordered systems, with flexible particles represented as collections of spheres (Calmettes *et al.*, 1993 ▶) or more commonly using an ensemble of molecular conformations (Ozenne *et al.*, 2012 ▶). Ensemble representations of such *supertertiary structure* (Tompa, 2012 ▶) were initially developed for NMR studies (Bernadó *et al.*, 2005 ▶), but were quickly extended to other techniques in structural biology including solution scattering. The *Ensemble Optimization Method* (*EOM*) (Bernadó *et al.*, 2007 ▶) was the first ensemble-based fitting strategy proposed to address the structural characterization of IDPs by SAXS, and a number of other implementations of this approach have followed *e.g.* MES (Pelikan *et al.*, 2009 ▶), BSS-SAXS (Yang *et al.*, 2010 ▶), EROS (Różycki *et al.*, 2011 ▶), ENSEMBLE (Krzeminski *et al.*, 2013 ▶). These SAXS-driven ensemble modelling programs have enabled effective characterization of a number of IDPs and modular single-chain proteins that was not possible by MX, NMR and microscopy alone. This success is now pushing development of the method for even more challenging studies in *unstructural biology*. These include complexes of protein and nucleic acids, mixtures of oligomeric states and assemblies, specification of surface contacts and interfaces, and symmetry operations. This work presents an enhanced version, *EOM* 2.0, introducing new developments that aim to expand the utility of the approach for solution scattering studies. Some limitations of the original implementation are addressed, and case studies where the prototypal version of *EOM* 2.0 has been successfully employed are discussed. Further, we analyse the capacity of the ensemble approach in SAXS to resolve distinct conformational states and also provide quantitative metrics for characterizing the results provided by the ensemble fitting.

## 2. The ensemble concept in SAXS   

When a solution of non-interacting chemically identical particles is illuminated in a SAXS experiment, the recorded scattering pattern *I*(*s*) is a sum of that produced by each particle averaged over all orientations, where *I* is the scattered intensity and *s* refers to the magnitude of the scattering vector defined as *s* = 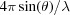 (where 2θ is the scattering angle and λ is the wavelength of the incident X-rays). For a system of flexible macromolecules, each conformation of each particle will contribute on the timescale of the SAXS measurement (typically, from sub-seconds to minutes). As the particles in a dilute system scatter independently, the time average due to changing conformation of a single particle is equivalent to the average over the entire ensemble. As a result, *I*(*s*) represents the sum of the average scattering intensities of all conformations present in the conformationally polydisperse solution (Svergun *et al.*, 2013 ▶). Thus the scattering data themselves will encode the degree of structural order/disorder. Qualitative assessment of structural disorder *versus* compactness of *e.g.* proteins can often be achieved by a transformation of the experimental data to a Kratky representation [*s* ^2^ *I*(*s*) *versus s*] (Glatter & Kratky, 1982 ▶), or using a dimensionless plot (*sR*_g_)^2^ *I*(*s*)/*I*(*0*) *versus sR*_g_, where *R*_g_ is the radius of gyration (Durand *et al.*, 2010 ▶). In these simple but informative plots globular macromolecules follow asymptotic behaviour at high scattering angles and display a bell-shaped peak with a well defined maximum at low angles. For disordered protein chains and polymers the peak at low angles is absent and an increase in intensity to a plateau followed by a monotonic increase at higher angles is observed [see reviews for details (Receveur-Brechot & Durand, 2012 ▶; Bernadó & Svergun, 2012 ▶). A distinct lack of features in the real-space distance-distribution function, *P*(*r*), computed by a Fourier transformation of *I*(*s*) (Bernadó, 2010 ▶) as well as the absence of a plateau in a Porod–Debye representation [*s* ^4^ *I*(*s*) *versus s* ^4^] (Rambo & Tainer, 2011 ▶) may also indicate disorder/flexibility. Additionally, failure to generate a satisfactory single model during rigid-body refinement against the experimental scattering data from a potentially flexible system may additionally suggest conformational polydispersity (Petoukhov *et al.*, 2012 ▶). In such cases an ensemble approach to modelling is likely to be more appropriate and may yield greater insight into the biology of the system. In such cases, an ensemble of conformationally polydisperse particles is approximated as a mixture according to equation (1):
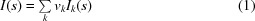
where *I_k_*(*s*) is the scattering intensity from the *k*th component and *v_k_* the volume fraction for that component (Konarev *et al.*, 2006 ▶). For flexible systems, the deconvolution of the scattering pattern into those from single components is clearly impossible given the very large number of conformers and an indirect approach is required. The strategy on which *EOM* is based consists of three main steps: (i) generate a large pool of possible conformations in order to approximate the (otherwise infinite) conformational space; (ii) compute the scattering profile for each conformation; (iii) select a subset of conformations that minimizes the discrepancy χ^2^ [equation (2)]:
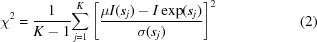
where *I*_exp_(*s*) is the experimental scattering, *K* is the number of experimental points, σ(*s_j_*) are standard deviations and μ is a scaling factor (Bernadó *et al.*, 2007 ▶).

*EOM* employs a genetic algorithm (GA) to select subsets of conformations from the random pool that best fit the experimental data. This selected ensemble then represents a low-resolution sample space which is used to generate distributions of structural parameters, and it is these distributions of parameters and not the ensemble members themselves that form the basis of the analysis. Results are then reported for the selected solutions as distributions of the parameters *R*_g_ and the maximum particle dimension (*D*_max_). These parameters are pre-calculated in real space from all conformers at the stage of pool generation. The distributions are then compared with those derived from the initial pool, representing the unrestricted conformational freedom of the system, in order to visually delineate overall properties of biomolecules such as *compactness* and *flexibility* (Bernadó *et al.*, 2007 ▶).

The widespread use of the original implementation of the *EOM* software and the subsequent adaptation of ensemble approaches in biological scattering studies demonstrate the usefulness of the method. However, the studies conducted have highlighted a number of limitations of SAXS-driven ensemble fitting and its applicability that necessitate further development for the extension of the method to more complicated cases. Also to be addressed is the often non-trivial interpretation of the results by visual comparison of the *R*_g_ and *D*_max_ distributions. An unambiguous approach based on widely shared metrics must also be introduced to enhance the fidelity of the ensemble method. These concerns are addressed in the following sections.

## 3. Advanced *EOM* 2.0: enhanced ensemble generation, selection and optimization   

The successful use of an ensemble approach to describe a solution of biomacromolecules rests on the following: (i) realistic and adequate sampling of conformational space during pool generation; (ii) the employment of an appropriate search technique (usually Monte Carlo-based) for the selection of an ensemble that optimally describes the experimental data; (iii) the presentation of results in a clear, straightforward and, whenever possible, quantitative way. The pool generation task must produce feasible models (*e.g.* avoiding steric clashes and maintaining chain connectivity) that cover the possible conformational space and, if available, incorporate high-resolution information from complementary techniques, *e.g.* MX and NMR. The search procedure should also be able to optimize the number of conformations in the ensemble. It should be noted here that the best strategy may not necessarily lie in the minimal number of conformations that can describe the experimental data (this principle is hardly applicable to very flexible systems like IDPs). Instead, a possibility should be provided to adjust the number of conformers in the search for the optimized ensemble contributing to the final scattering. These considerations form the basis for the developments discussed below.

### 3.1. Intelligent generation of missing fragments   

SAXS is commonly employed to account for mobile regions absent from high-resolution structures. Often these regions are disordered and must be modelled taking into account particular features of unstructured and/or flexible macromolecules (*e.g.* dihedral angles). For the ensemble generation as applied to proteins, a heuristic algorithm for intelligent browsing of the bond *versus* dihedral angle distribution represented by the C_α_–C_α_ Ramachandran plot (Kleywegt, 1997 ▶) (Fig. S1 in the supporting information) has been implemented. This approach allows missing regions or ‘linkers’ to be feasibly modelled without size limitations, using a *random* or *native* sequence designation for the generation of ‘fully disordered’ and ‘less disordered’ regions, respectively. Both modes generate the chains following Flory’s relationship [equation (3)] (Flory, 1953 ▶):
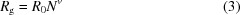
where *N* is the number of monomeric units in a polymer chain, *R*_0_ is a constant that depends on the persistence length of the polymer and *v* is an exponential scaling factor [for IDPs, *R*_0_ = 1.927±0.27 and *v* = 0.598±0.028 (Kohn *et al.*, 2004 ▶), and for chemically denatured proteins *R*_0_ = 2.54±0.01 and *v* = 0.522±0.01 (Bernadó & Svergun, 2012 ▶). The impact/robustness of the introduction of this feature is discussed below.

### 3.2. Oligomer generation and symmetry operations   

**Figure 1:**
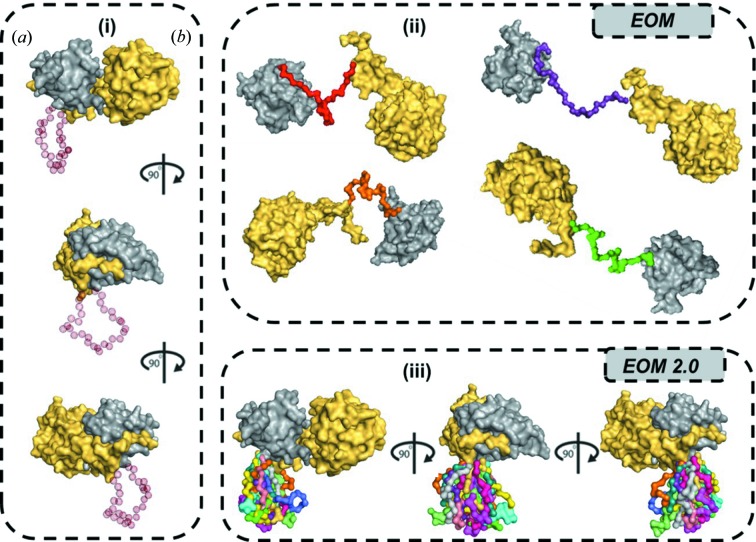
(i) Different views of an example multi-domain protein composed of two domains [solved by MX: (*a*) grey and (*b*) yellow], connected by a disordered linker 30 amino acids long (transparent red spheres, left area). (ii) Multiple inter-domain linker reconstructions (multiple colours) computed with *EOM* (upper-right area). (iii) Different views of multiple inter-domain linker reconstructions computed with *EOM* 2.0 using the new possibility to fix domain positions in three-dimensional coordinates (bottom-right area).

In the original *EOM* implementation, tools for oligomer generation and for the definition of specific interfaces and inter-domain/subunit contacts were not available. To overcome this shortcoming, the ability to fix all the subunits in defined positions and orientations is now introduced, significantly reducing the probability of obtaining nonsensical conformations and allowing for the generation of specific structural assemblies (Fig. 1 ▶).

**Figure 2:**
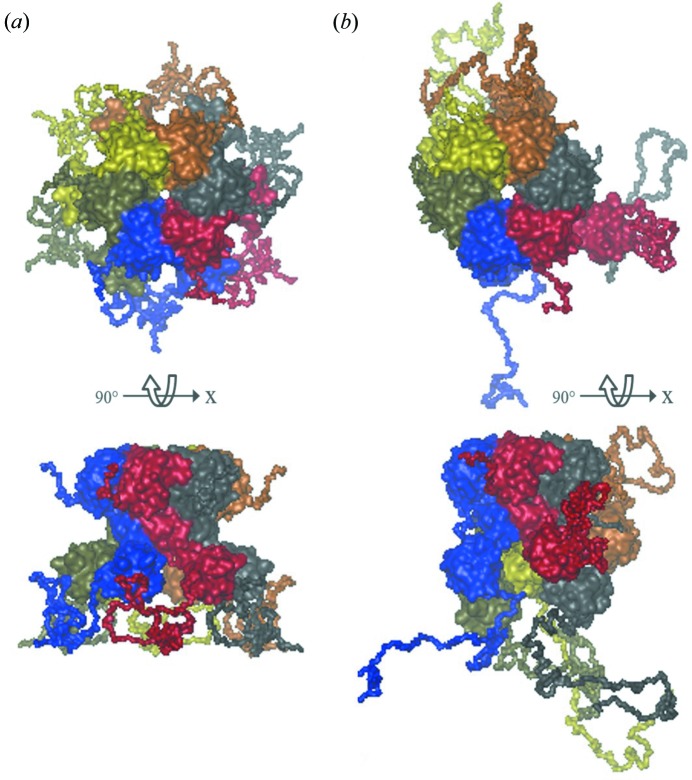
Different views of a hexameric multi-domain protein with a symmetric oligomeric core. Each monomer is composed of two domains connected by a flexible linker and with disordered N- and C-termini. (*a*) Generated full-length hexamer where *P*6 symmetry is applied to the core and to the disordered regions. (*b*) Asymmetric modelling where the generated chains are independent of each other and the symmetry is present in the core only.

Oligomeric assemblies with flexible regions are an important class of biological macromolecules, and their study adds an extra level of complexity to the ensemble-based modelling. Disordered regions in a multi-domain protein may retain the symmetry observed for the high-resolution core, and the generation of symmetric configurations of the full-length protein based on this core can help to significantly restrict, as well as better describe, the conformational space from which the search algorithm samples (Fig. 2 ▶ *a*). As symmetry may not necessarily be preserved beyond the core structure, it is also important that modelling asymmetric flexible sections is possible (Fig. 2 ▶ *b*). The options to import a high-resolution oligomeric core structure and the generation of user-defined interfaces have been introduced into *EOM* 2.0, with the latter option using a contact distance restraint between specified sequence positions. In cases where a definite oligomeric core structure is not available, complementary biophysical techniques and tools, *e.g. PISA* (Krissinel & Henrick, 2007 ▶), can be used to detect potential interfaces. Non-crystallographic symmetry (*i.e.* symmetry *P*7, *P*9, *P*11 *etc*.) can also be applied. The current implementation provides interface definitions and symmetry operations, allowing for the modelling of complex inter-particle interactions (*e.g.* protein–protein or protein–nucleic acids), while still broadly sampling the available conformational space and generating configurations free of steric clashes.

### 3.3. Optimization of the ensemble size   

In the original *EOM* it was assumed that each member of the selected ensemble contributes equally to the overall scattering intensity, with the ensemble size predefined (by default, 20 conformations). In the present version, the ensemble size may be optimized during the minimization procedure together with the selection of the conformers. The search procedure randomly selects ensembles within a customizable range of sizes, uniformly distributed. The ensemble that best minimizes the discrepancy χ^2^ is selected and its size represents the refined number of conformations, each with individual weights (fractions of occupancy). Accordingly, more flexible proteins are described by more populate ensembles (typically between ten and 20; up to a maximum of 50 conformers are allowed) whereas the scattering from more rigid molecules can still be fitted by a small number of conformations (between two and five but theoretically down to one, if there is a single conformer that provides an excellent fit). In other ensemble fitting approaches, weighting of particular conformers has been adopted, although implemented differently, and a minimal set of several dominant conformations was searched to describe the scattering data (Pelikan *et al.*, 2009 ▶; Yang *et al.*, 2010 ▶). Looking for a minimum set may provide good results for macromolecular systems adopting a few defined conformations, but this is not likely to be an optimum strategy for very flexible systems, such as IDPs, which adopt an astronomic number of configurations in solution.

### 3.4. Ensemble fitting with multiple pools   

SAXS-driven ensemble fitting can also be applied to the study of mixtures, including oligomeric equilibria and solutions of multiple distinct particle species. In such cases, when complementary methods provide additional information on the components and/or assemblies present, multiple pools can be generated for each species (*e.g.* monomer, dimer, tetramer). These pools can be obtained externally or generated by *EOM* 2.0, and compose an expanded search space. If warranted (*e.g.* based on analytical ultracentrifugation data), the percentage of models selected from each pool may be defined prior to optimization. Care must be taken with the interpretation of such an analysis as the systems containing multiple species are yet less determined compared to those with only conformational polydispersity. Supporting information from other sources is often required to draw meaningful conclusions from the analysis of multiple pools (see §5).

### 3.5. Measures of flexibility, *R*_flex_ and *R*_σ_   

The major result of *EOM* analysis are the distributions of low-resolution structural parameters (*R*_g_ and *D*_max_), which describe the flexibility of the system. These distributions are obtained by averaging multiple runs of the GA (Bernadó *et al.*, 2007 ▶) and encode information about the states assumed by the particles in solution. They can be described as probability density functions *S* = (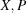), where *P* = (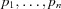) is the probability ascribed to the interval *X* = {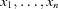} such that
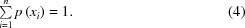
The characteristics of the selected ensemble are compared to those displayed by the pool allowing one to assess the flexibility of the system. Previously, decoding of this information was left to the visual perception of the user leaving room for potential misinterpretations; here we introduce a quantitative measure utilizing the concept of information entropy.

**Figure 3:**
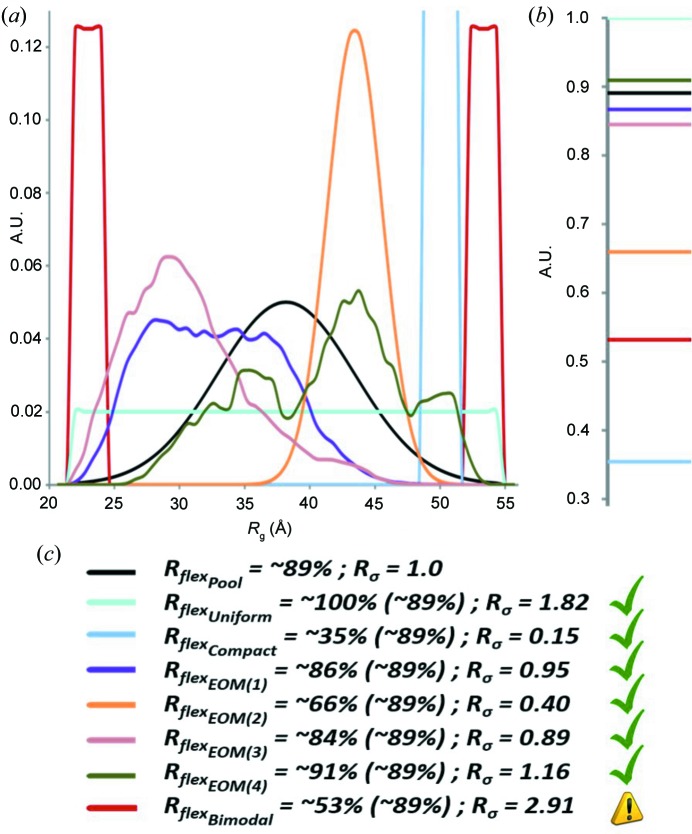
Qualitative characterization of particle flexibility from various characteristic *R*_g_ distributions. (*a*) Pool (black), which represents the case of complete randomness; *EOM*(1) (purple), *EOM*(2) (orange), *EOM*(3) (pink) and *EOM*(4) (dark green) which represent the real outcome of independent *EOM* 2.0 runs in terms of *R*_g_ distributions; uniform (cyan), compact (light blue), bimodal (red) which represent extreme (theoretical) cases. (*b*) *H*_*b*_(*S*) values computed from the distributions in (*a*). (*c*) Combination of *R*_flex_ values for all the distributions (and compared to the threshold of randomness computed from the pool, in brackets, ∼89%) with the associated *R*_σ_ values. The last example (red curve) indicates a potentially inconsistent result.

The entropy *H_b_*(*S*) (Shannon & Weaver, 1949 ▶) 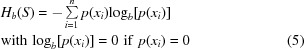
can be conveniently applied to enable a quantitative characterization of *EOM* size distributions (see the supporting information for further details). Indeed, a protein showing a broad Gaussian-like distribution of parameters, where it is assumed the disordered regions move randomly in solution, can be viewed as a carrier of high uncertainty. Here, *H_b_*(*S*) tends to −1, which is expected to be close to the *H_b_*(*S*) calculated for the pool. Conversely, a protein with a narrow size distribution (a scenario where the particle exhibits limited flexibility) provides low uncertainty, with *H_b_*(*S*) tending to 0. Consequently, the distributions, *i.e.* uniform [*H_b_*(*S*) = −1] and single value [*H_b_*(*S*) = 0], are then considered as representations of extreme, albeit theoretical, cases of maximal flexibility and complete rigidity, respectively. The information content, or *entropy*, can therefore be used as a quantitative measure of flexibility (Figs. 3 ▶ *a*, 3 ▶ *b*) with a metric we define as *R*_flex_ 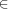 [0,1]: 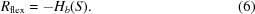
Using *R*_flex_, the selected ensemble distribution can be numerically compared to that of the pool, the latter representing a reference for flexibility. For convenience, *R*_flex_ can be reported as a percentage in the range 0 to 100%, with *R*_flex_ = 100% indicating maximum flexibility. This convention will be followed here.

The *R*_flex_ metric allows one to quantify the difference between flexible and rigid systems; it is especially useful in conjunction with the additional metric *R*_σ_:
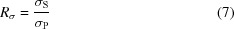
where σ_S_ and σ_P_ are the standard deviations for the distributions of the selected ensemble and of the pool, respectively. *R*_σ_ indicates the variance of the ensemble distribution with respect to the original pool, yielding values close to 1.0 when the ensemble distribution describes a fully flexible system and largely reproduces the conformational space of the pool. Therefore, in cases where *R*_flex_ is smaller than that of the pool distribution, *R*_σ_ should be below unity. Conversely, when *R*_flex_ tends to values greater than that of the random pool, *R*_σ_ > 1.0. For the cases where *R*_flex_ is significantly smaller than that of the random distribution but *R*_σ_ > 1, further investigation is required as this combination may point towards poor data quality. The above-mentioned cases (Fig. 3 ▶ *a*) are therefore compared in Fig. 3 ▶(*c*) where conclusions regarding ensemble flexibility based on the *R*_flex_ and *R*_σ_ metrics are reported, allowing also an automated check for discovering potential artifacts *e.g.* due to poor data quality or aggregation. In addition to *R*_flex_ and *R*_σ_, distributions are also compared using a set of standard descriptors: *standard deviation*, *average absolute deviation*, *kurtosis*, *skewness* and *geometric average* (see the supporting information).

## 4. Applications of *EOM* 2.0: tests and case studies   

The major prerequisites for successful application of the SAXS-driven ensemble-based modelling are: (i) the initial search pool is populated with models that describe well the available conformational space, and (ii) that a robust ensemble selection is driven appropriately by the experimental data. The following tests and case studies demonstrate that the new implementation of *EOM* does successfully meet these conditions.

### 4.1. Adequate sampling of the conformational space of unfolded proteins   

The population of end-to-end distances of unfolded proteins is expected to follow that of a Gaussian distribution, with a mean squared end-to-end distance equal to 70(± 15) Å^2^ × *N*, where *N* is the number of amino acids in the polypeptide chain (Fitzkee & Rose, 2004 ▶). It is thus important to ensure that the members of a search pool generated for unfolded proteins conform to this behaviour.

**Figure 4:**
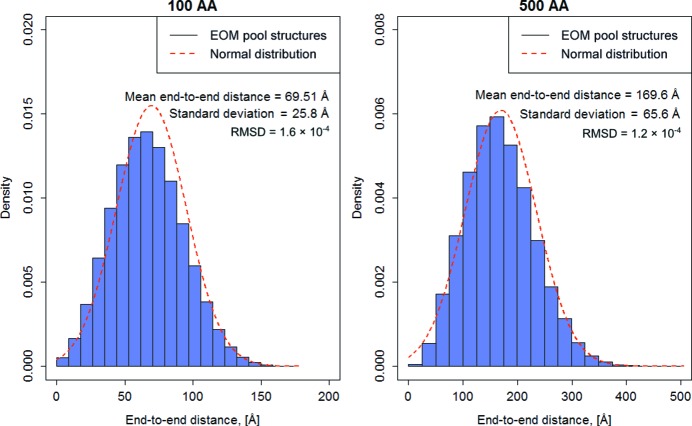
Distribution of end-to-end distances computed from pools containing 10 000 structures of 100 and 500 amino-acid chains and compared with the expected normal distribution having the same mean and standard deviation values.

In the first test of *EOM* 2.0 for unfolded proteins, pools of 10 000 polyalanine models of sequence length 100 or 500 amino acids were generated in *random* mode. This process was repeated five times for each sequence length and the end-to-end distances for individual models were calculated using the statistical package *R* (R Core Team, 2014 ▶). Histogram plots of each pool were then generated and compared to the theoretical Gaussian distributions calculated using the same mean and standard deviation values as extracted from the pool (Fig. 4 ▶). In both cases the resulting distributions well approximate the theoretical Gaussian with averaged RMSDs (between densities of normal and pool distributions) of 1.6 × 10^−4^ (*N* = 100) and 1.2 × 10^−4^ (*N* = 500), respectively. The mean squared end-to-end distances were 5591 Å^2^ (∼56 × *N*, *N* = 100) and 33 057 Å^2^ (∼66 × *N*, *N* = 500) for smaller and larger chains, respectively, in agreement with the experimental data for unfolded proteins (Tanford *et al.*, 1966 ▶). This result indicates that a pool of 10 000 conformations is sufficient to approximate the conformational space of unfolded proteins.

### 4.2. Evaluation of amino-acid number-to-*R*_g_ ratio   

The radius of gyration, *R*_g_, is one of the most important parameters for the analysis of macromolecules by SAXS. In *EOM* 2.0, generation of disordered regions is based on an intelligent usage of the *native* or *random* dihedral angle modes derived from the C_α_–C_α_ Ramachandran plot. The capability of the method to generate feasible models that satisfy the theoretical *R*_g_ values described in §3.1 for IDPs and chemically denatured proteins was therefore tested for both *native* and *random* modes.

According to Flory’s equation [equation (3)] the *R*_g_ of a peptide chain is power-law dependent on the number of amino acids. To investigate the concordance of *EOM* 2.0 with this expectation, multiple pools (15) of 10 000 conformers of polyalanine were constructed in *random* as well as *native* modes. This was repeated for sequences of varying length (10, 20, 50, 100, 200 amino acids) and the average *R*_g_ was calculated. Fig. S3 shows the agreement of the average *R*_g_ for the pools generated in both modes with the theoretical estimations of *R*_g_ using equation (3). Thus search pools generated in both modes by *EOM* 2.0 well represent chemically denatured proteins as well as IDPs. Moreover, the *a posteriori*-extracted parameter *v* is in agreement with the theoretical values found in the literature (Fig. S4).

### 4.3. Discrimination between distinct conformations   

*EOM* is often employed for the analysis of flexible multi-domain particles, especially proteins, for which high-resolution fragment structures are available (*i.e.* domains, subunits, ligands). These modular systems often provide regulatory mechanisms by adopting specific subsets of conformations in solution. For example, *open* and *closed* protein conformations can expose or hide important surface residues essential for interaction with specific or generic partners, with conformational flexibility the key driver of the function. SAXS-driven ensemble fitting approaches are well suited to the study of these systems, providing the means to examine the degree of flexibility and to discriminate between the dominant conformations present under specific solution conditions.

**Figure 5:**
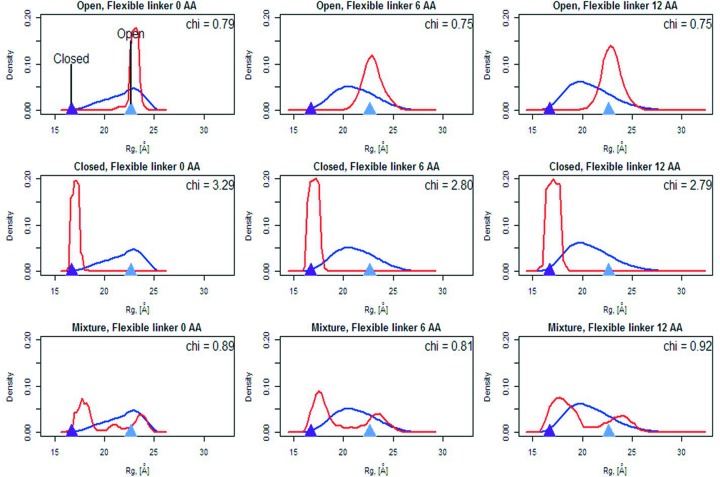
Distributions of *R*_g_ pools (blue) and selected ensembles (red) for determination of open and closed conformations of calmodulin using three different lengths of inter-domain disordered linkers (zero, six and 12 amino acids) for the pool generation. Violet and light blue triangles show *R*_g_ for closed and open conformations, respectively.

The high-resolution structures of the protein calmodulin, a Ca^2+^-binding protein with a myriad of cellular functions dependent on its conformational state, were used to test the power of *EOM* 2.0 to discriminate between *open* [PDB entry 1cll (Chattopadhyaya *et al.*, 1992 ▶), *R*_g_ = 22.6 Å] and *closed* [PDB entry 1ctr (Cook *et al*., 1994 ▶), *R*_g_ = 16.7 Å] conformations. As calmodulin has two well defined EF-hand domains connected by a labile helical linker, it provides an excellent case to test the capacity of *EOM* 2.0 to identify dominant conformational states from an ensemble of configurations. The theoretical scattering intensities of the open and closed forms of calmodulin were calculated using *CRYSOL* (Svergun *et al.*, 1995 ▶), and the theoretical scattering intensity of a mixture of *open* and *closed* conformations (no intermediate conformations are here considered) simulated by averaging the intensities of the open and closed forms using *PRIMUS* (Konarev *et al.*, 2003 ▶). Three pools of 30 000 conformations were generated, each with a different flexible sequence length between the two EF-hand domains (zero, six or 12 amino acids). The theoretical SAXS profiles of calmodulin corresponding to the open, closed and mixture states were used as input for *EOM* 2.0 and the ability of the program to identify the dominant conformations tested. The resulting distributions of *R*_g_ presented in Fig. 5 ▶ show that in all cases the genetic algorithm was able to resolve the two conformations (*i.e. open versus closed*), demonstrating that *EOM* 2.0 has sufficient discriminating power for the successful resolution of distinct conformations from a solution mixture.

### 4.4. *EOM* resolution and multimodal distributions   

**Figure 6:**
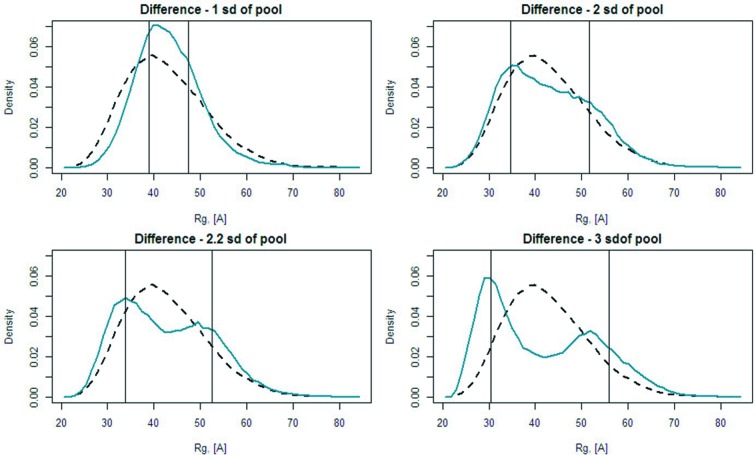
Comparison of *R*_g_ distributions showing that subpopulations of conformers can be identified from a large ensemble if the difference between their mean *R*_g_ is greater than approximately two times the standard deviations of the original pool (bottom left). The *R*_g_ values of the two subpopulations are indicated as vertical lines on each plot.

The question of the ‘resolution’ of *EOM* is clearly important, especially when dealing with multimodal distributions. In a bimodal distribution of *R*_g_, for example, what is the minimum difference between the two dominant *R*_g_ peaks or ‘subpopulations’ that can be distinguished? To test the resolution of *EOM* 2.0, an initial pool (10 000 models) of polyalanine (either 100 or 500 amino acids) was generated and a subset of conformers representing two subpopulations, each with a different mean *R*_g_ and standard deviation, extracted. The theoretical scattering intensities of the members of the subset were calculated by *CRYSOL* and averaged, producing a simulated test data set. These data were then used as input for selection against another independently generated pool (10 000 models of either 100- or 500-residue polyalanine), and the resulting selected size distributions were examined. The test was repeated several times varying the difference between the mean *R*_g_ of each subpopulation and also their standard deviations. The *R*_g_ distributions produced from these tests demonstrate the resolution capability of *EOM* 2.0, where the bimodal distributions expected are indeed observed, indicating that subpopulations of structures can be resolved (Fig. S5). The results show that the resolution does not depend on the width (standard deviation) of the subpopulations, up to the point of intersection, but strongly depends on the absolute difference in the value of mean *R*_g_. As shown in Fig. 6 ▶, two subpopulations should show a relative difference greater than approximately two times the standard deviation of the pool from which they come in order to be distinguished. This result did not depend on the number of amino acids in the protein.

### 4.5. Robust ensemble fitting and the impact of noise   

Any method based on fitting experimental data with generated models is subject to the presence of experimental errors. To test the robustness of *EOM* 2.0 to random noise, the following simulations were conducted: (i) a pool of 10 000 polyalanine structures (100 amino-acid residues) was generated and five models with *R*_g_ close to the mean of the pool (29.05 Å) were selected. The theoretical scattering curves of the selected models were computed with *CRYSOL* and averaged. The scattering curve was then modified by adding random noise with the magnitude from 0 to 20% of the intensity values in order to simulate errors and varying data quality in experimental data (Fig. S6A). The procedure was repeated 50 times and all these simulated scattering curves were used as input for *EOM* 2.0. The same initial pool was used for the genetic algorithm as the software should provide similar solution, irrespective of the noise level. Strikingly, even at a high noise level (up to 20%) and with a good fit, the average *R*_g_ for the final ensemble solution is found to be in very good agreement with the *R*_g_ computed from the Guinier region (Fig. S6B). This remarkable stability of the average *R*_g_ is not surprising if one considers that the value is effectively determined using the information from the entire scattering pattern, and not just from the Guinier region. It can therefore be concluded that *EOM* 2.0 is able to provide reliable solutions up to a 20% noise level in the experimental data.

## 5. Discussion   

Techniques and strategies targeting unstructured systems such as IDPs and flexible modular proteins are increasingly used by researchers. Understanding how structural disorder relates to function requires modification of approaches in structural biology, as such systems are not, for example, readily crystallized for MX studies. Ensemble-based methods have been introduced for the characterization of flexible biological macromolecules and have been shown to be a highly useful addition to the tools employed in *unstructural biology* (Tompa, 2011 ▶). The main goal of the present work was the enhancement of the capability of a widely used ensemble-based SAXS approach for the characterization of flexible particles. The original *EOM* was applied frequently since its release in 2007 (over 370 citations as to September 2014), yielding interesting results and new insights for numerous flexible systems. The functional limitations of the approach have also been highlighted by the active user community, providing the impetus for further development of the method.

In the present work we describe an enhanced *EOM* 2.0, which has been redesigned in a modular format allowing also for further modifications and future developments. An artificial intelligence approach for the generation of *ad hoc* flexible linkers has been introduced, enabling an adequate pool generation, and tools for the generation of multi-subunit assemblies with defined interfaces, symmetry and flexible linker regions were introduced. These improvements overcome the limitations of the original implementation, where only single-chain particles could be modelled and symmetry was not considered. *EOM* 2.0 has already been successfully employed in a prototypal study of the flexible, trimeric protein gephyrin (Sander *et al.*, 2013 ▶), where the new multi-subunit capabilities facilitated the first structural study of this dynamic protein in solution.

A question often asked of ensemble-based methods concerns the number of conformations required to represent the selected ensemble. In *EOM* 2.0 an *optimal* ensemble size can be automatically determined during the selection procedure driven by the genetic algorithm. This new optimization reduced potential overfitting as shown by the application of both *EOM* and *EOM* 2.0 to the work of Soykan *et al.* (2014 ▶) where a point mutation of the enzyme collybistin (CB_SH3+_^E262A^) favours the flexibility of its SH3 domain and thus the synaptogenic activity of collybistin in recruiting the partner protein gephyrin. The ensemble of runtime-optimized number of conformations provided by *EOM* 2.0 yields similar results with a smaller number of conformers compared to that of the user analysis with a manually defined number of conformations (Fig. S7). Generally, the fewer conformers selected by *EOM* 2.0, the more rigid the protein; flexible systems still need dozen(s) of conformers in the optimized ensemble. The automated selection of the number of conformers is an alternative to the approaches implemented by other groups, *e.g.* minimal set (Pelikan *et al.*, 2009 ▶) or jackknife procedure jointly using NMR and SAXS data (Sterckx *et al.*, 2014 ▶).

*EOM* 2.0 makes it possible to combine multiple independently generated pools, expanding the search space of the method. This capability was critical in the study of an oligomeric mixture of full-length mitochondrial glutaminase C (Møller *et al.*, 2013 ▶), where *EOM* 2.0 was used to search simultaneously through three different search pools (dimer, tetramer and octamer). The results obtained explain how the disordered regions of this protein influence the distribution of oligomeric states and thus the enzymatic activity. This study provided the first ever experimentally derived structural model of the full-length mitochondrial glutaminase C, highlighting the crucial role played by the disordered regions in keeping the enzyme tetrameric – which corresponds to the active state.

The results of the original *EOM* were analysed through visual inspection of the distributions. Structural features were identified qualitatively by the user and the discrimination of significant differences between distributions was performed interactively. Here, two metrics facilitating a quantitative measure of flexibility have been introduced, *R*_flex_ and *R*_σ_, complementing the low-resolution structural descriptors, *R*_g_ and *D*_max_. The combined use of the new metrics provides a powerful tool for the automatic detection of potential artifacts that may lead to spurious conclusions. The utility of the combined *R*_flex_ and *R*_σ_ is well illustrated in cases where the distributions of selected ensembles are multimodal. For example, an extreme bimodal distribution (Fig. S2B, red), interpreted naively based only on visual inspection, may lead to the conclusion that the system has maximum possible flexibility. If this is indeed the case, *R*_flex_ of both the random pool and the selected ensemble will be similar (with *R*_flex_ approaching 100%). However, if the naive conclusion is wrong, *R*_flex_ of the selected ensemble will be significantly lower than that of the pool (*R*_flex_ of the selected ensemble lower than ∼50%) suggesting a reduced flexibility.

**Figure 7:**
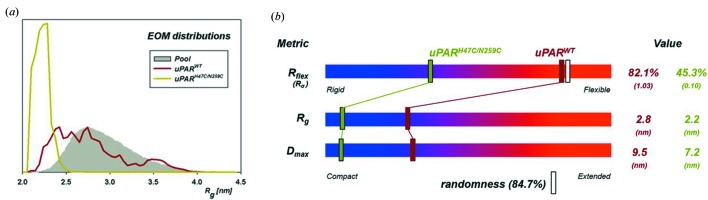
Characterization of the flexibility of uPAR^WT^ and the mutated uPAR^H47C-N259C^ using *EOM* 2.0. (*a*) Size distributions (*R*_g_) of uPAR^WT^ and uPAR^H47C-N259C^, providing only a qualitative assessment through direct comparison of the distributions of the selected ensembles and the pool. (*b*) The metrics *R*_flex_ and *R*_σ_ enable characterization of the flexibility quantitatively, with *R*_flex_ = ∼82% and *R*_flex_ = ∼45%, for uPAR^WT^ and uPAR^H47C-N259C^, respectively, reflecting a significant change in compactness of the particle upon mutation (with a threshold of randomness of ∼85% calculated from the pool).

The application of *R*_flex_ and *R*_σ_ to reanalyse the data of the multi-domain urokinase-type plasminogen activator receptor (uPAR) (Mertens *et al.*, 2012 ▶) demonstrates the suitability of these new metrics for systematic studies of domain flexibility. In this study the recalcitrant uPAR was investigated by solution SAXS due to an inability to crystallize the receptor, suggesting that the domains of uPAR may be flexible and hinting that perhaps such flexibility may also drive function. The analysis demonstrated that the wild-type receptor was indeed flexible, compared with a stabilized mutant [for which the crystal structure of a closed conformation could be determined (Xu *et al.*, 2012 ▶)]. Using the metric *R*_flex_ the flexibility of wild-type uPAR – previously only qualitatively assessed (Fig. 7 ▶ *a*) – is quantified as *R*_flex_ = ∼82% suggesting a nearly random conformation of the linkers (with a threshold of randomness derived from the pool of ∼85%). Conversely, the cysteine-bridged mutant of the receptor, uPAR^H47C-N259C^, shows significantly reduced flexibility quantified by an *R*_flex_ = ∼45% (Fig. 7 ▶ *b*).

*EOM* 2.0 has been extensively tested in order to demonstrate the benefits as well as identify the limitations of its use. *Random* coils as well as more ordered/*native* structures conforming to theoretical expectation in terms of *R*_g_ can be generated and pools of 10 000 conformations have been shown to well approximate the entire conformational space (otherwise infinite) for very flexible particles such as IDPs. The capability and limitations of *EOM* 2.0 to discriminate between open/closed conformations have also been studied. The program with the manual can be freely downloaded as part of the *ATSAS* package from http://www.embl-hamburg.de/biosaxs/software.html and the users may post queries to the discussion forum http://www.saxier.org/forum/viewforum.php?f=10.

## 6. Conclusions   

*Ensemble Optimization Method*, *EOM* 2.0, for the characterization of flexible systems using SAXS in solution has been redesigned, and its application range broadened to oligomeric systems as well as macromolecular complexes. In addition, metrics for quantitative measurement and identification of flexibility have been introduced facilitating quantitative and systematic analysis of the studies of flexible macromolecules in solution.

Physical simulation methods using molecular dynamics and energy minimization to predict macromolecular trajectories are providing more and more impact in the modelling of flexible systems (*e.g.* Banavali & Roux, 2011 ▶; Brewer *et al.*, 2011 ▶; Das & Pappu, 2013 ▶; Luan *et al.*, 2014 ▶). Consequently, a lively discussion has emerged on the relations between experimentally derived ensembles and those predicted by physical simulations (Jensen & Blackledge, 2014 ▶; Wang *et al.*, 2014 ▶). *EOM* 2.0 can easily be used for bridging SAXS, computational methods or other experimental techniques given the possibility to use any external pool (*e.g.* generated with molecular dynamics, based on NMR or others) in the ensemble selection. The developments presented here should therefore enhance the role of solution SAXS as an essential structural method to be synergistically coupled with complementary high-resolution techniques in the study of flexible particles.

## Supplementary Material

Supporting information. DOI: 10.1107/S205225251500202X/fc5007sup1.pdf
