# Supplementary material for: Advanced ensemble modelling of flexible macromolecules using X-ray solution scattering
Source: IUCrJ. 2015 Feb 26;2(Pt 2):207–17. doi: 10.1107/S205225251500202X (PMC4392415; doi:10.1107/S205225251500202X)
Supplement: Supplementary file 1 [file m-02-00207-sup1.pdf]

IUCrJ

Volume 2 (2015)

Supporting information for article:

Advanced ensemble modelling of flexible macromolecules using X-ray solution scattering

Giancarlo Tria, Haydyn D. T. Mertens, Michael Kachala and Dmitri I. Svergun

S1. Information entropy

According to information theory the amount of information content carried by a distribution is measured in terms of *entropy* (Shannon and Weaver 1949). In the Shannon theory, the *entropy* $H_b(S)$ is adopted as a measure of the information carried by *P* over *X* with higher *entropy* corresponding to more uncertainty (less information). Broad (e.g. Gaussian distributed) phenomena result in higher entropy (less information) whereas populations distributed over a small variance with respect to the average have lower uncertainty (more information). The entropy is calculated according to the Shannon formula (Eq. S1):

$$H_b(S) = -\sum_{i=1}^{n} p(x_i)\log_b(p(x_i)) \qquad \text{(S1)}$$

$$\textit{with} \ \log_b(p(x_i)) = 0 \ \ \textit{if} \ \ p(x_i) = 0$$

where $H_b(S)$ Є [-1,0] with *b* representing the *'cardinality of the alphabet'* in the original approach proposed by Shannon in communication theory (for simplicity $b = |X|^i$ in EOM 2.0). The randomness of observed phenomena can thus be quantified. Gaussian-like distributed phenomena will show $H_b(S)$ → -1 as they carry little information. Conversely, observations clustered over a restricted number of intervals x_i carry higher information with $H_b(S)$ → 0. Furthermore, the two extreme cases of a uniform distribution where all intervals (events) *X* have the same probability *P(X)* (no information at all) and a narrow distribution with all the data concentrated in a single interval x_i with $P(x_i) = 1$ (maximal information) will have $H_b(S)$ = -1 and $H_b(S)$ = 0 respectively.

S2. Descriptors

In EOM 2.0, five statistics are computed and shown along with the distributions with the aim to compare the pool and the final solution. These descriptors are: *(1) standard deviation, (2) average absolute deviation, (3) kurtosis, (4) skewness, and (5) geometric average*.

$$(1) = \sqrt{\frac{1}{n-1}\sum_{i=1}^{n}\left(x_i - \bar{x}\right)^2} \qquad (2) = \frac{1}{n}\sum_{i=1}^{n}\left|x_i - m(X)\right| \qquad (3) = \frac{\frac{1}{n}\sum_{i=1}^{n}\left(x_i - \bar{x}\right)^4}{\left(\frac{1}{n}\sum_{i=1}^{n}\left(x_i - \bar{x}\right)^2\right)^2} - 3$$

$$(4) = \frac{\frac{1}{n}\sum_{i=1}^{n}\left(x_i - \bar{x}\right)^3}{\left(\frac{1}{n}\sum_{i=1}^{n}\left(x_i - \bar{x}\right)^2\right)^{3/2}} \qquad (5) = \sqrt[n]{\prod_{i=1}^{n} x_i}$$

(1) **Standard deviation:** measures the amount of variation or dispersion from the average.

(2) **Average absolute deviation:** measures the average of the absolute deviations and is a summary statistic of statistical dispersion or variability. Normal distributions are expected to show an average absolute deviation ~0.8 times the standard deviation.

(3) **Kurtosis:** measures the "*peakedness*" of a probability distribution.

(4) **Skewness:** measures the asymmetry of a probability distribution about its mean.

(5) **Geometric average:** measures the central tendency of a set of numbers

References

Bernado, P. and D. I. Svergun (2012). "Structural analysis of intrinsically disordered proteins by small-angle X-ray scattering." Mol Biosyst **8**(1): 151-167.

Guillou, J. C. L. and J. Zinn-Justin (1977). "Critical Exponents for the n-Vector Model in Three Dimensions from Field Theory." Phys. Rev. Lett. **39**(95).

Kohn, J. E., et al. (2004). "Random-coil behavior and the dimensions of chemically unfolded proteins." Proc Natl Acad Sci U S A **101**(34): 12491-12496.

Shannon, C. E. and W. Weaver (1949). The Mathematical Theory of Communication, Univ. of Illinois Press, Urbana, IL.

Soykan, T., et al. (2014). "A conformational switch in collybistin determines the differentiation of inhibitory postsynapses." EMBO J.

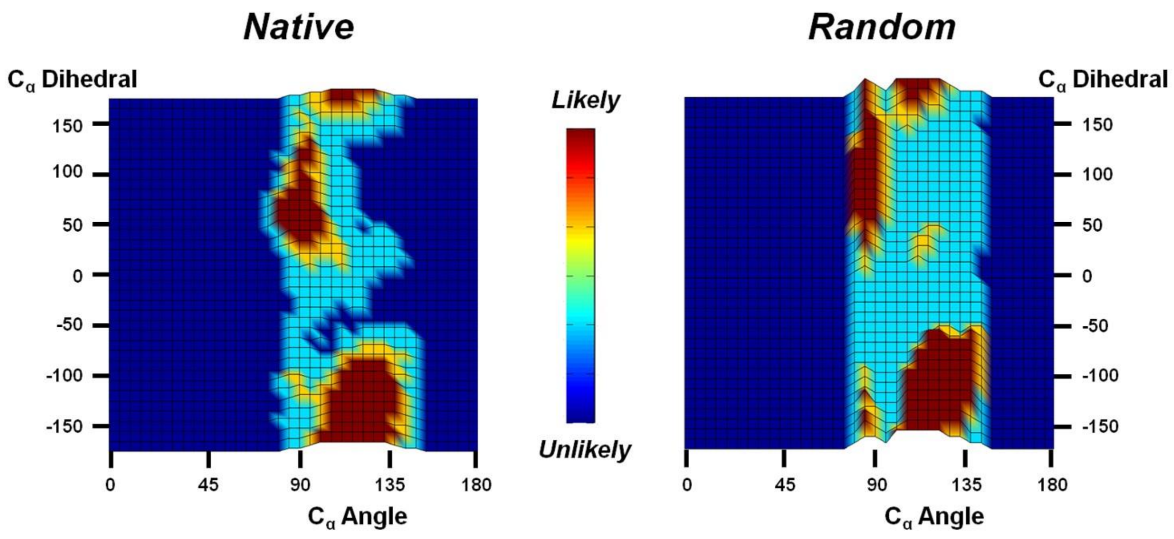

Figure S1 Distribution of C_α backbone bond vs. dihedrals angles for *native* (left) and *random* (right) generation. Maps for the *native* and *random* C_α-C_α distributions where constructed from selected PDB entries according to (Kleywegt 1997), using the entire sequence for the *native* case and unstructured sequence regions for the *random* case to represent completely denatured proteins or random coils.

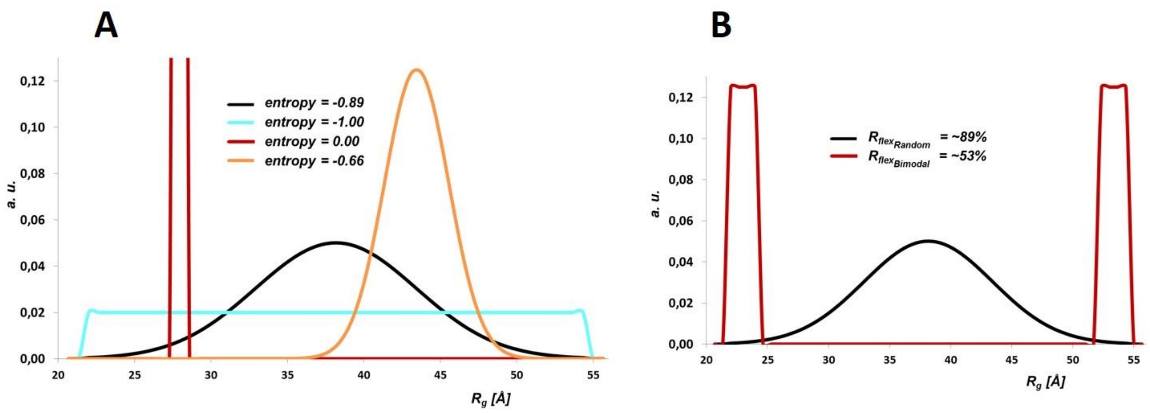

Figure S2 Graphical illustration of the usage of entropy as measure of flexibility. (A) Measure of the entropy for theoretical distributions. (B) Extreme theoretical case where the use of entropy alone would lead to a wrong interpretation of the flexibility.

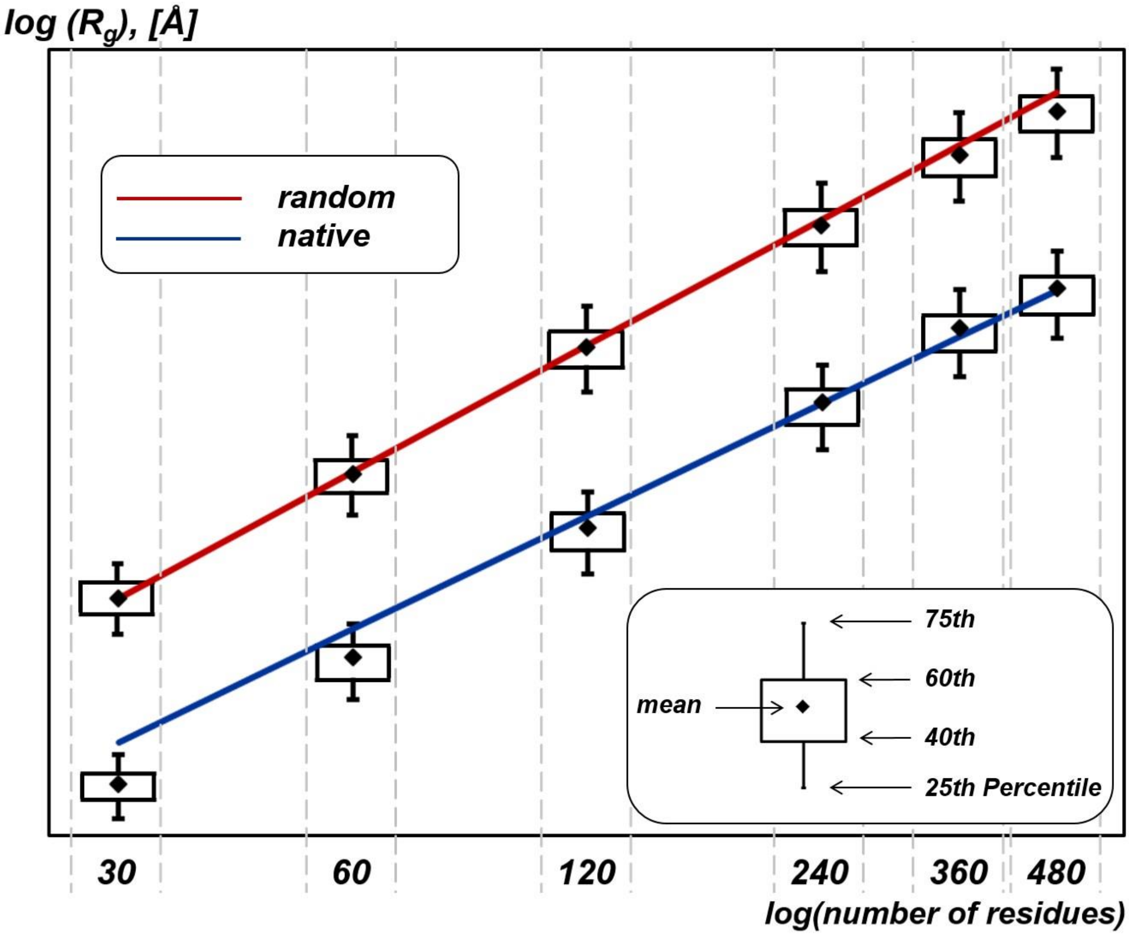

Figure S3 Dependence of R_g values (represented as Whiskers box) extracted from pools of models generated using EOM 2.0 (in *random* and *native* modes) and the theoretical R_g expectations based on Flory's relationship using the parameters $R_0 = 1.927$ and $v = 0.598$ in the case of chemically denatured proteins (Kohn et al. 2004) (red), and $R_0 = 2.54$ and $v = 0.522$ for intrinsically disordered proteins (Bernado and Svergun 2012) (blue).

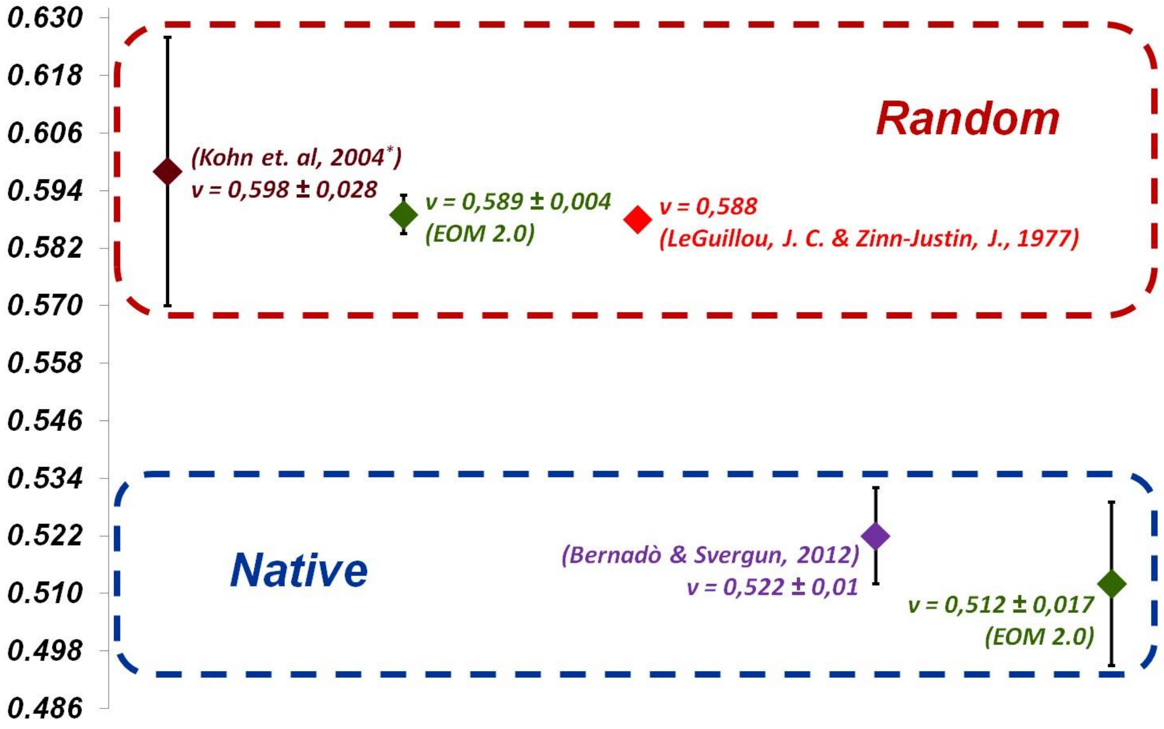

Figure S4 Comparison of the scaling factor parameter *v* (Flory's relationship) extracted from the models generated by using EOM 2.0 with the values extracted theoretically (Guillou and Zinn-Justin 1977) as well as experimentally (Kohn, Millett et al. 2004) and for *random* (upper red section) and native (lower blue section) conformations respectively (Bernado and Svergun 2012).

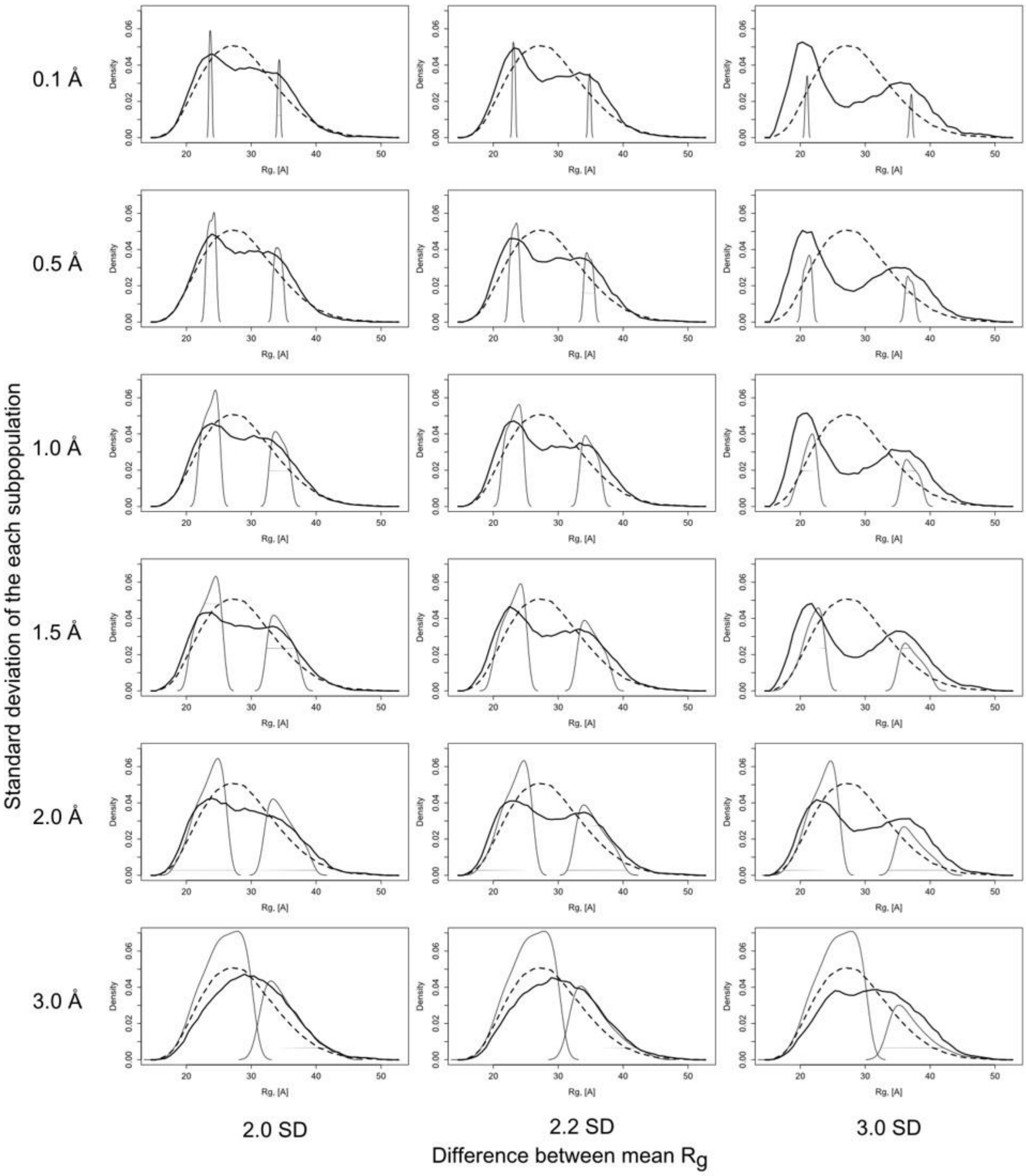

Figure S5 Distribution of pools (*black dashed lines*) and selected ensembles (*blue solid lines*) with various standard deviations between mean R_g of the subpopulations (*grey solid lines*). The comparison shows that the EOM 2.0 resolution does not depend on the width (standard deviation) of subpopulations, unless they intersect, but on the absolute difference between their mean R_g.

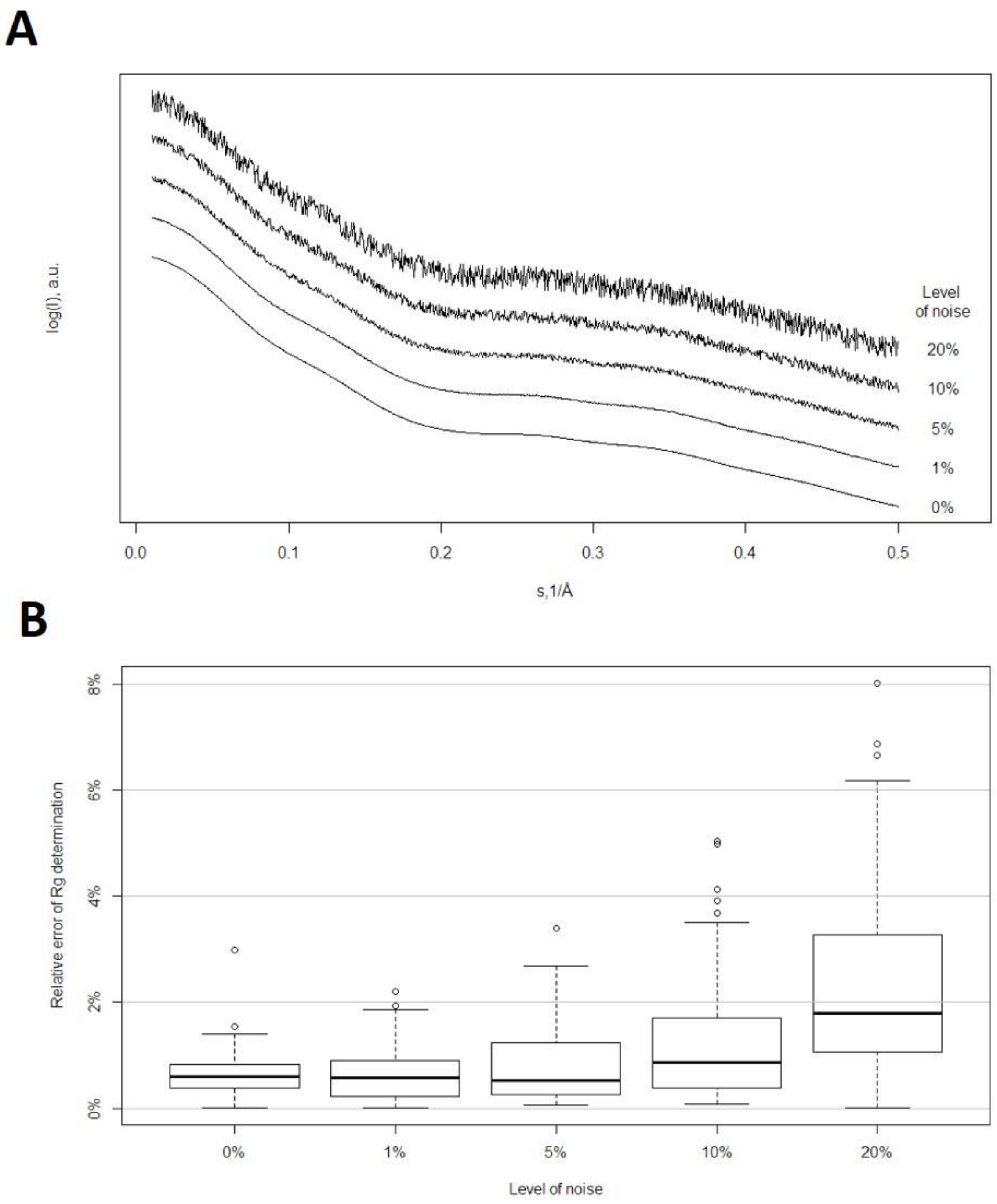

Figure S6 (A) Comparison of scattering curves used to check the robustness to noise of EOM 2.0 in the case of complete absence of noise (0%) and with 1%, 5%, 10% and 20% random noise respectively. (B) Dependence of relative error in the R_g determination on level of noise.

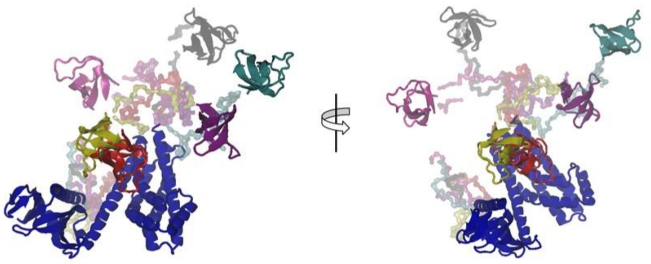

	R_g	D_{max}	(%)
Purple	34.41	117.92	4%
Cyan	40.93	148.16	24%
Black	44.14	159.33	12%
Yellow	31.08	100.03	12%
Red	30.25	102.94	20%
Magenta	37.52	116.52	28%
Total	***36.79***	***124.61***	***100%***

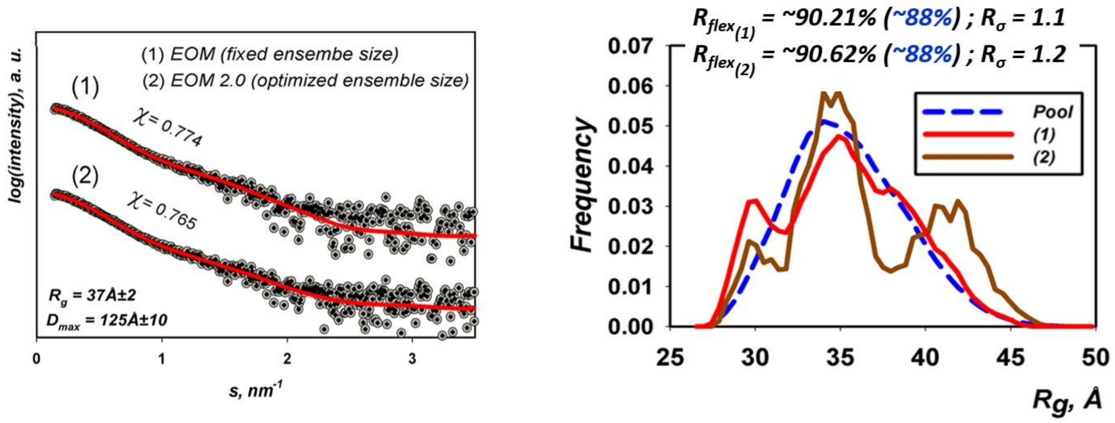

Figure S7 Summary of the application of both EOM and EOM 2.0 to the study of (Soykan, Schneeberger et al. 2014). *(up)* The optimized ensemble search procedure implemented in EOM 2.0 automatically defines the number of conformations that composes the solution ensemble. The models (8 for this specific case) are presented as cartoon using different colours. *(bottom-left)* Experimental scattering data and theoretical fit computed using both EOM and EOM 2.0. *(bottom-right)* Quantitative measurement of the flexibility (by using R_{flex} and R_{σ}) does not indicate significant difference when compared to that derived from the original implementation. Moreover, the improved resolution of EOM 2.0 allows to better identify three main subpopulation present in the ensemble.

---

[i] Usually |X|=54 in EOM 2.0, with 50 intervals effectively used for the distribution and 4 used for smoothing
